# Hospital and Health News

**Published:** 1923-02

**Authors:** 


### Hospital and Health News.

This year the Metropolitan Hospital Saturday Fund celebrates its jubilee.

Prince Henry has accepted the presidency of Queen Mary's Hospital, Stratford.

Dr. John Weir has been appointed Physician-in-Ordinary to the Prince of Wales.

The undergraduates of Birmingham University raised by their Christmas carnival £3,000 for the hospitals.

A new X-ray department is to be provided at Sheffield Royal Infirmary, and a tender of £1,650 has been accepted.

A sum of £32,000 has been raised by the carnival and bazaar on behalf of the Royal Liverpool Children's Hospital.

Deaths from cancer in Kent during 1921 were the highest on record, and almost 50 per cent. more than the figures for 1908.

Many of the letters from Canada posted during December bore the post-mark : " Christmas seals : Stamp out Tuberculosis."

The first six months' working of the workmen's penny a week contribution scheme have produced £130 for Dorking Hospital.

A site has been obtained in Peel Street for the proposed new hospital for women at Nottingham, the building of which is to begin as soon as possible.

It is reported that negotiations are in progress for the purchase of the Foundling Hospital, on the site of which shops, blocks and cinemas will be built.

Messrs. J. & A. Churchill have published, at 2s. 6d., an eighth edition of " A Code of Rules for the Prevention of Infectious and Contagious Diseases in Schools."

Viscount Lascelles has become Vice-President of the Royal Bath Hospital, Harrogate, of which since it was founded in 1824 the Earls of Harewood have been patrons.

A design for a statue of Lord Lister, by Mr. G. H. Paulin, A.R.S.A., has been accepted by the Lister Memorial Fund, which it is hoped to place in Kelvingrove Park, Glasgow.

A second edition of " The Birth and Early Days of our Ambulance Trains in France, August, 1914," by Colonel G. A. Moore, C.M.G., D.S.O., M.D., has been issued by Messrs. John Bale & Danielsson.

The Feltham District Council has asked the Ministry of Health to hold a public inquiry into the administration of Staines Joint Isolation Hospital, in consequence of certain complaints that have been made.

The late Sir James Ormiston Affleck, M.D., has left £8,000 to the Edinburgh Royal Infirmary, and £5,000 to the Royal Edinburgh Hospital for Incurables. At the former, his will records, " a large part of my life's work has been done."

Alderman G. Claridge Druce, M.A., LL.D., has resigned the chairmanship of the Public Health Committee of the City of Oxford, which he has held for twenty-six years. He now takes the chairmanship of the Parliamentary Committee.

The Darlington Joint Hospital and War Memorial Committee have £52,000 in hand, apart from the value of the existing institution for rebuilding. Plans have been prepared by Mr. Edwin Hall, and Messrs. Clark & Moscrop, of Darlington.

Queen Mary's Hospital for the East End has received £10,564 from its appeal. The total was announced at the recent Festival Dinner by Major Jackson, who has succeeded Mr. Scrivener in the post of secretary, that the latter had filled for twenty-two years.

It is announced that the Southwark Board of Guardians has decided to supply butter to the nurses of Dulwich Hospital, where an outbreak of diphtheria occurred lately, and it is also suggested that butter should be served to all inmates over 60 years of age. A patient, a sister, and a probationer contracted the disease, and at one time as many as thirteen nurses were off duty.

Ashburton (Devon) Urban Council received no reply to their advertisement for a medical officer. The salary offered was £25 a year.

Dr. Norman Macfadyen has resigned the position of Medical Officer of Health for Letchworth, as he is seeking fuller public service.

The Rotherham Hospital has decided not to combine with the penny in the pound put forward by the Sheffield Hospitals Joint Committee.

The Supreme Court in Cape Town lately awarded damages to a patient who had not given his consent to a serious operation performed on him in a public hospital.

A block of two storeys, with 40 additional beds, is being added to Sherburn Hospital, on the Scotch border. The architect is Mr. John Hall, F.R.I.B.A., of Sunderland.

Willing's Press Guide.—Messrs. Willing have issued the 1923 (jubilee number) of this most valuable book of reference. As usual, it has been carefully revised and brought up to date, and is a mine of ready information on all matters concerning the Press.

The "Mission Hospital" formerly known as " Mercy and Truth," is a useful monthly record of the medical missions of the Church Missionary Society, a valuable form of mission work that is too little known. The articles and illustrations describe medical work in out-of-the-way places.

Enlarging Poplar Hospital.—Mr. J. Oatley is the architect of the extensions at Poplar Hospital, which include new quarters for nurses, a casualty unit, massage and electrical rooms, and the lowering of the present ground floor. The cost of the alterations is expected to be £30,000.

Lectures on Tuberculosis and Venereal Disease.—A course of lectures on Tuberculosis and Venereal Disease is being delivered in the Lecture Hall of the Royal Institute of Public Health, 37 Russell Square, W.C.1, on Wednesdays, at 4 p.m., from January 17 to March 21. No tickets of admission are required.

Help for Diabetes Research.—The sum of £400, to be paid annually for six years, has been given to Guy's Hospital by Messrs. Ashton & Parsons, Ltd., to be spent on research into diabetes mellitus. A Parsons Fellowship has thus been founded. Under the Medical Research Council, the hospital has been already at work.

Closing Woolwich Arsenal Hospital.—The decision to close the Arsenal Hospital, Woolwich, has been an unwelcome surprise to the district. Accidents are to be sent to the Herbert Hospital ; diseases to the Auxiliary Hospital, and two qualified first-aid men remain on the spot. The staff in the Arsenal is about 6,000, and their work is necessarily dangerous.

Lectures on Infant Care and Management.—A course of lectures on Infant Care and Management is being given at the Infants' Hospital, Vincent Square, on Wednesdays, at 6 p.m. from January 10 to March 28. Though intended primarily for the nurses of the hospital outsiders are welcomed, and tickets can be obtained from the Secretary of the Hospital—5s. for the course, or 1s. for any individual lecture.

Measuring the Height of Children.—Speaking at King's College to women health visitors and school nurses on the " Health of the School Child," Dr. D. C. Kirkhope, Medical Officer of Health for Tottenham, said that a change in the method of measuring the height of school children will shortly be introduced. The child's sitting height will in future be taken as the standard, the length of the body being of infinitely greater importance than the length of the legs.

Enlarging Yeovil Hospital.—The new building of the Yeovil and District Hospital has been finished, and work began in Christmas week without formal ceremony. Since extension of the original hospital was impracticable, Kingston Manor House and grounds were purchased for £3,500, and the first half of the original scheme, costing £18,000, has now been finished. The new hospital provides for over fifty patients and has rooms for private cases.

The War on Rats.—Since February, 1901, when the City Corporation first undertook the work of systematically exterminating rats in ships and warehouses in the Port of London, 1,250,691 have been destroyed.

Nourishing Qualities of "Long Pig."—Lecturing on "Vitamines" at the Exhibition of Scientific Novelties at King's College, Professor Winifred Callis, while repudiating the idea that the proper food of man is man, said that experiments on animals showed that we could do with less protein if we fed on human flesh rather than on any other flesh known.

Trained Nurses in Korea.—At the last meeting of the Occidental Graduate Nurses' Association of Korea, which is composed of all the Missionary nurses in the country, united action was taken to supply funds to finance the printing of a text-book on nursing which is being translated by one of the Missionary nurses—nine of the nurses making themselves responsible for finding the money. Steps were also taken to organise a Graduate Nurses' Association among the Koreans.

New Scottish Health Minister.—Mr. James Kidd has resigned the office of Parliamentary Under-Secretary for Health for Scotland, and the Secretary for Scotland has appointed Captain Walter E. Elliot, M.C., M.P. Captain Elliot has represented the Lanarkshire Division of Lanark since 1918. He had a distinguished military career, serving continuously from August 4, 1914, until the end of the war, for the greater part of the time as medical officer to the Scots Greys. He won the Military Cross with bar.

Dangerous Drugs.—The Home Office has issued during the month a memorandum with reference to the position of doctors and dentists under the Dangerous Drugs Act of 1920. The drugs excluded from the operation of the Act are specified and the best means of inspection explained. This inspection is to be carried out in England and Wales by Medical Officers of Health of the Ministry of Health, and in Scotland by Medical Officers of the Scottish Board of Health, who will have the right of entry to premises without giving any previous warning. The memorandum is about to be published in pamphlet form by the Stationery Office.

Doctors Renounce the Telephone.—All the doctors in the Coalville district of Leicestershire have removed the telephone from their houses and surgeries because they say they have often been rung up for trifling reasons. This has led to so many complaints that the Leicestershire Health Insurance Committee has protested to the doctors on their panel that the removal of the telephones is likely to lead to less effective medical service than is desired by the Committee. Protest that the absence of telephonic communication to the doctors may sometimes prolong suffering is also being made by the Coalville Friendly Societies' Council, representing about 30 societies and nearly 7,000 members.

New Year Knighthoods.—The list of New Year's Honours includes knighthoods for William Heaton Hamer, M.A., M.D., F.R.C.P., Medical Officer of Health for London; Bernard Henry Spilsbury, M.A., M.B., B.Ch.(Oxon), Hon. Pathologist to the Home Office ; David Drummond, M.A., M.D., Vice-Chancellor of Durham University, President and Professor of Medicine, University of Durham College of Medicine ; Norman Walker, M.D., L.L.D., Chairman of the Consultative Council for Medical and Allied Services of the Scottish Board of Health; and Thomas Edwin Cooper, F.R.I.B.A., Architect of the Port of London Authority's new buildings and Architect to the College of Nursing.

The Journal of Clinical Research.—The first post-war number of the "Journal of Clinical Research" (Watergate House, York Buildings, Adelphi, W.C.), the official organ of The Clinical Research Association, has been issued at the price of 1s. "Since the original appearance of the Journal," writes the Editor, "profound changes have occurred in almost every branch of clinical pathology. It is inevitable that, between physician and pathologist, a far closer link should nowadays be necessary. Therefore, although the Journal will continue to provide an epitome of the quarter's scientific advances and literature, yet its pages must also encourage true and personal co-operation between the Members and Pathologists of the Association." Correspondence columns are opened and space set aside for Notes and Queries.

### VOLUNTARY HOSPITALS COMMISSION.

At a meeting of representatives of local voluntary hospital committees held in July last, it was decided to adopt a proposal made by the Voluntary Hospitals Commission to establish a small consultative committee of members of local committees. The Commission, after inviting nominations from local hospital committees, have now appointed the following :—

The Right Hon. F. D. Acland, Devonshire ; Alderman E. C. Barnes, C.B.E., Derbyshire ; Captain W. Best, North Wales ; Mr. J. J. Cockshott, O.B.E., Lancashire ; The Rev. G. B. Cronshaw, Berks, Bucks and Oxon ; Mr. H. N. Crouch. Somerset ; Sir James Curtis, K.B.E., Birmingham and Gloucestershire ; Mr. Walter Davies, Manchester ; Sir David Drummond, C.B.E., Northumberland ; Mr. H. W. C. Drury, Norfolk ; Sir Henry Hadow. C.B.E., Sheffield ; Mr. Noel Hanbury, C.B.E., Hampshire ; Viscount Lascelles, K.G., D.S.O., East and West Ridings of Yorkshire ; Colonel R. J. S. Simpson, C.B., C.M.G., Kent ; Sir A. Garrod Thomas, Monmouthshire ; Bishop Welldon, Durham.

